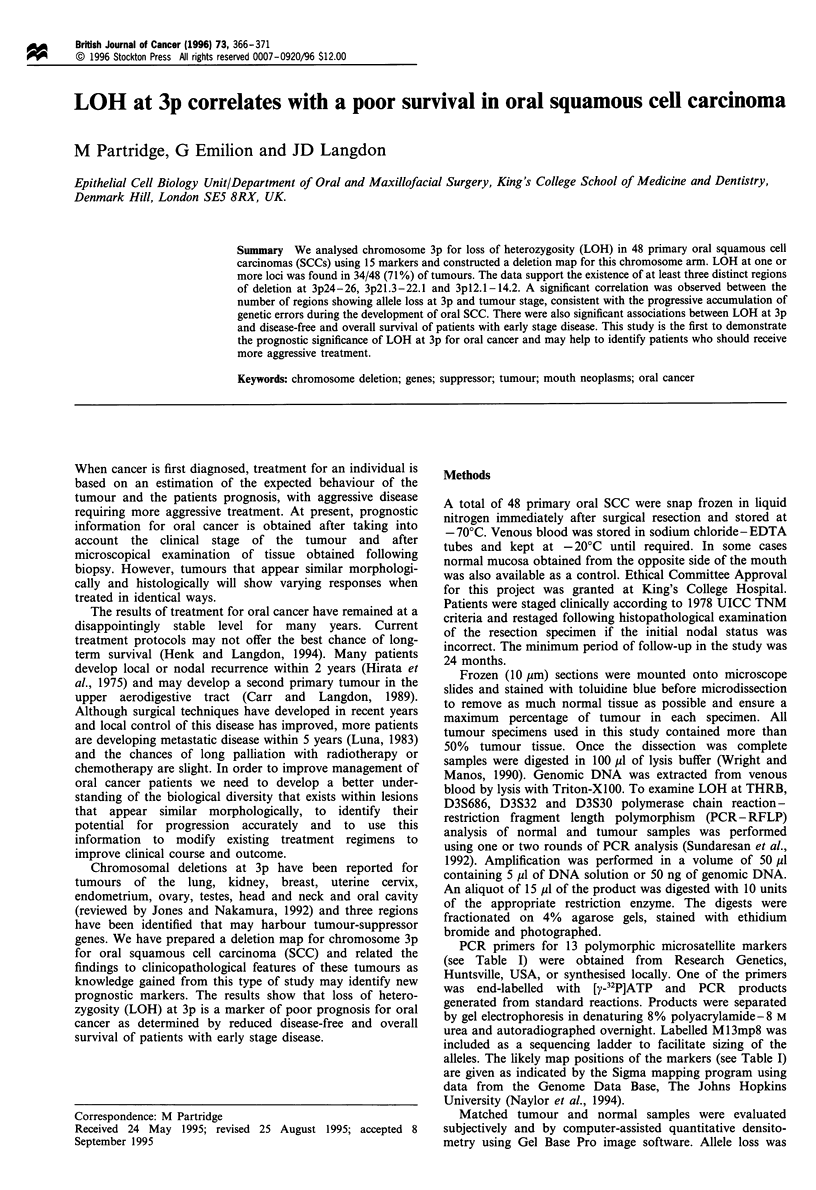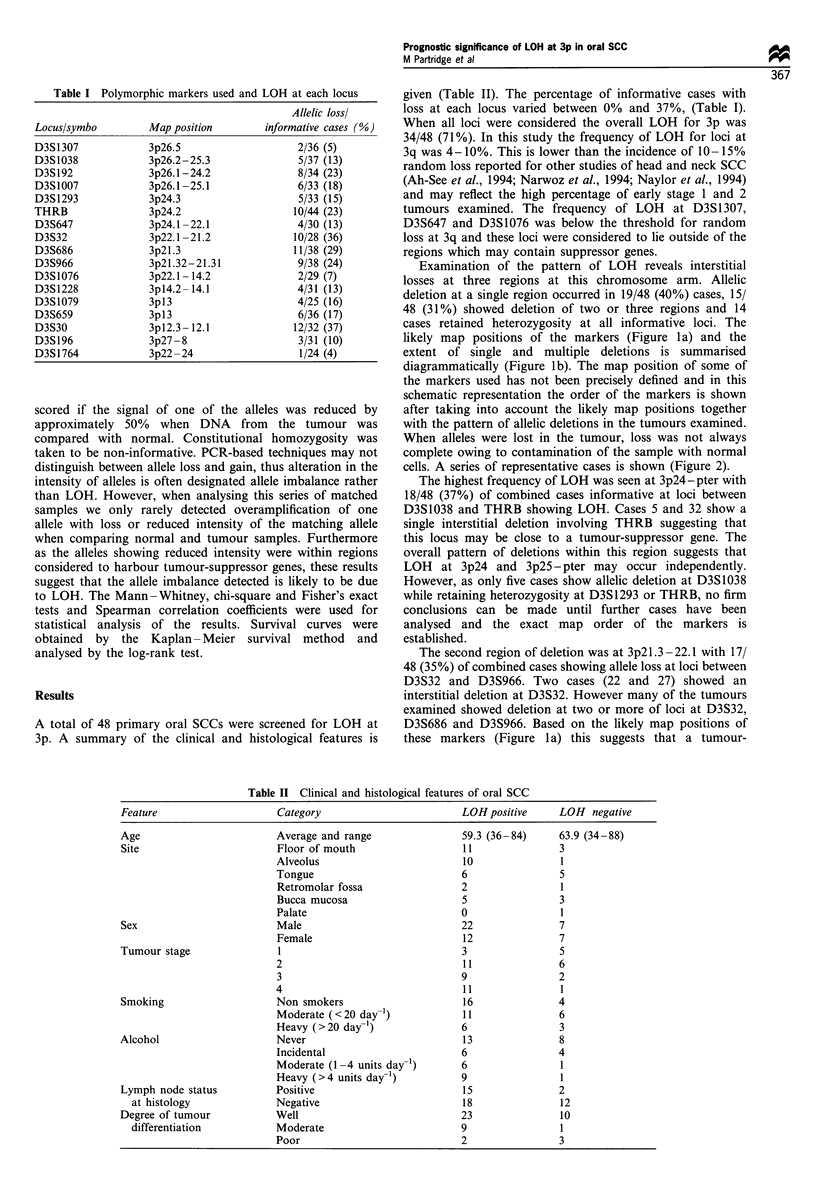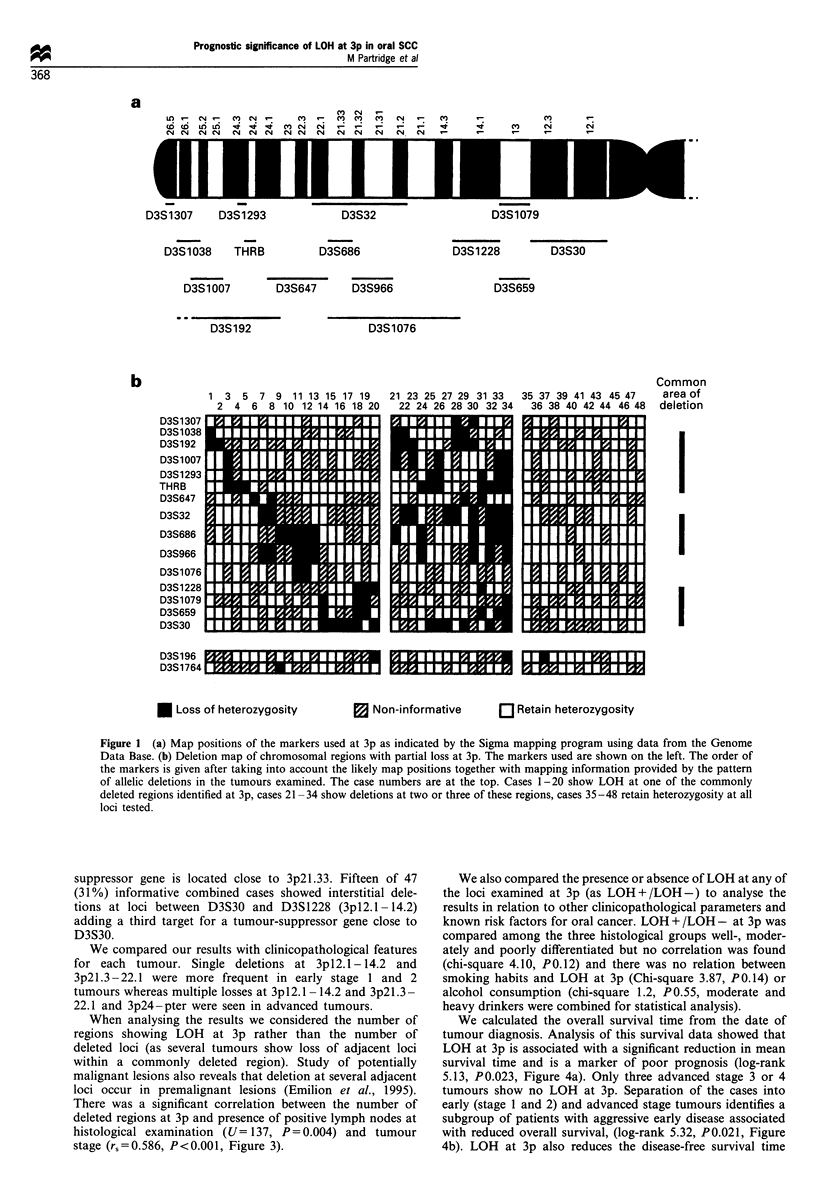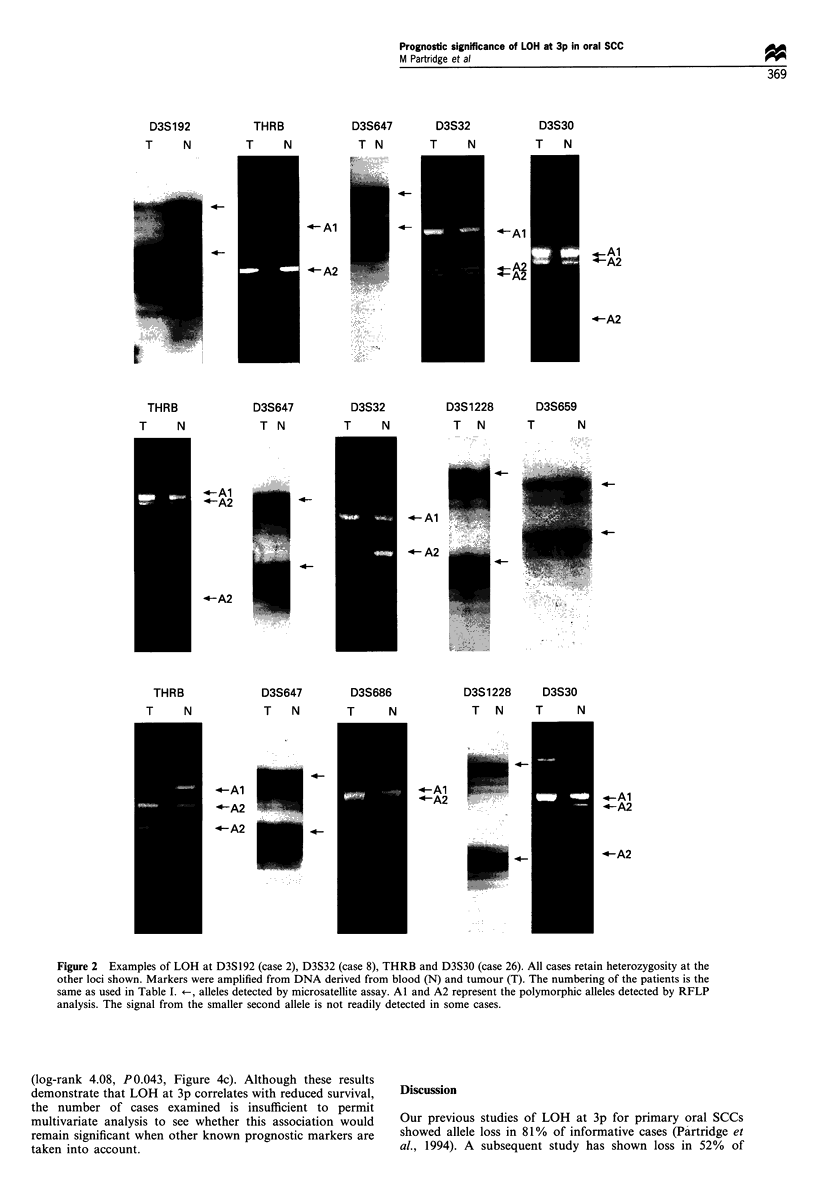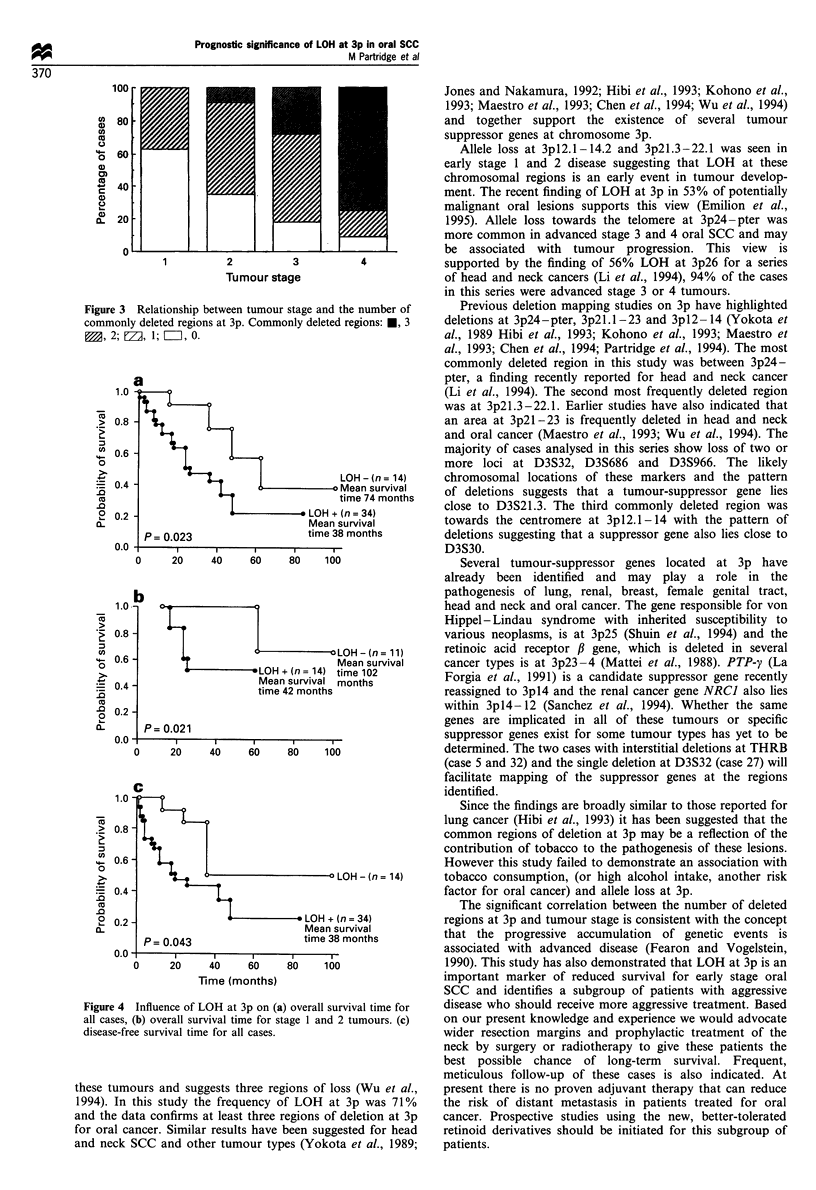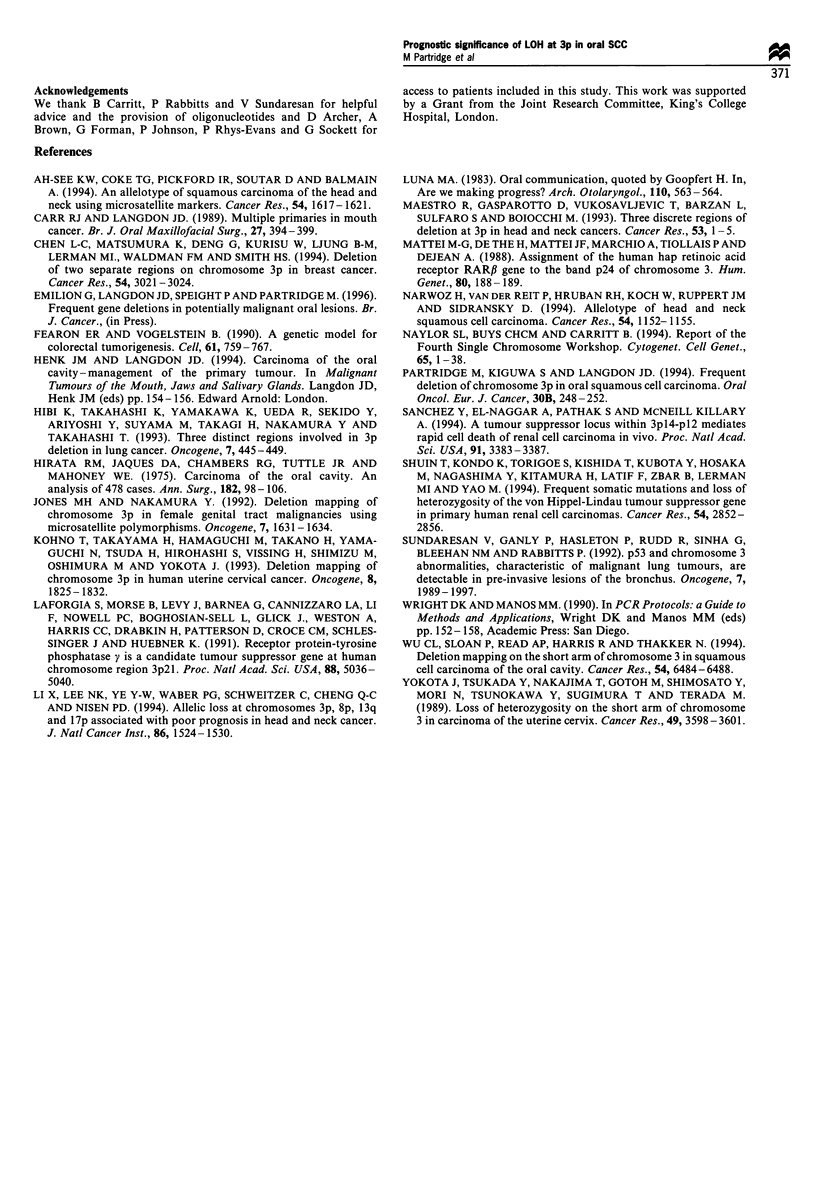# LOH at 3p correlates with a poor survival in oral squamous cell carcinoma.

**DOI:** 10.1038/bjc.1996.62

**Published:** 1996-02

**Authors:** M. Partridge, G. Emilion, J. D. Langdon

**Affiliations:** Epithelial Cell Biology Unit, Department of Oral and Maxillofacial Surgery, King's College School of Medicine and Dentistry, London, UK.

## Abstract

**Images:**


British Journal of Cancer (1996) 73, 366-371

© 1996 Stockton Press All rights reserved 0007-0920/96 $12.00

### LOH at 3p correlates with a poor survival in oral squamous cell carcinoma

M Partridge, G Emilion and JD Langdon

*Epithelial Cell Biology Unit/Department of Oral and Maxillofacial Surgery, King's College School of Medicine and Dentistry, Denmark Hill, London SE5 8RX, UK.*

**Summary** We analysed chromosome 3p for loss of heterozygosity (LOH) in 48 primary oral squamous cell carcinomas (SCCs) using 15 markers and constructed a deletion map for this chromosome arm. LOH at one or more loci was found in 34/48 (71%) of tumours. The data support the existence of at least three distinct regions of deletion at 3p24-26, 3p21.3-22.1 and 3p12.1-14.2. A significant correlation was observed between the number of regions showing allele loss at 3p and tumour stage, consistent with the progressive accumulation of genetic errors during the development of oral SCC. There were also significant associations between LOH at 3p and disease-free and overall survival of patients with early stage disease. This study is the first to demonstrate the prognostic significance of LOH at 3p for oral cancer and may help to identify patients who should receive more aggressive treatment.

**Keywords:** chromosome deletion; genes; suppressor; tumour; mouth neoplasms; oral cancer

When cancer is first diagnosed, treatment for an individual is based on an estimation of the expected behaviour of the tumour and the patients prognosis, with aggressive disease requiring more aggressive treatment. At present, prognostic information for oral cancer is obtained after taking into account the clinical stage of the tumour and after microscopical examination of tissue obtained following biopsy. However, tumours that appear similar morphologically and histologically will show varying responses when treated in identical ways.

The results of treatment for oral cancer have remained at a disappointingly stable level for many years. Current treatment protocols may not offer the best chance of long-term survival (Henk and Langdon, 1994). Many patients develop local or nodal recurrence within 2 years (Hirata *et al.*, 1975) and may develop a second primary tumour in the upper aerodigestive tract (Carr and Langdon, 1989). Although surgical techniques have developed in recent years and local control of this disease has improved, more patients are developing metastatic disease within 5 years (Luna, 1983) and the chances of long palliation with radiotherapy or chemotherapy are slight. In order to improve management of oral cancer patients we need to develop a better understanding of the biological diversity that exists within lesions that appear similar morphologically, to identify their potential for progression accurately and to use this information to modify existing treatment regimens to improve clinical course and outcome.

Chromosomal deletions at 3p have been reported for tumours of the lung, kidney, breast, uterine cervix, endometrium, ovary, testes, head and neck and oral cavity (reviewed by Jones and Nakamura, 1992) and three regions have been identified that may harbour tumour-suppressor genes. We have prepared a deletion map for chromosome 3p for oral squamous cell carcinoma (SCC) and related the findings to clinicopathological features of these tumours as knowledge gained from this type of study may identify new prognostic markers. The results show that loss of heterozygosity (LOH) at 3p is a marker of poor prognosis for oral cancer as determined by reduced disease-free and overall survival of patients with early stage disease.

Correspondence: M Partridge
Received 24 May 1995; revised 25 August 1995; accepted 8 September 1995

### Methods

A total of 48 primary oral SCC were snap frozen in liquid nitrogen immediately after surgical resection and stored at −70°C. Venous blood was stored in sodium chloride-EDTA tubes and kept at −20°C until required. In some cases normal mucosa obtained from the opposite side of the mouth was also available as a control. Ethical Committee Approval for this project was granted at King's College Hospital. Patients were staged clinically according to 1978 UICC TNM criteria and restaged following histopathological examination of the resection specimen if the initial nodal status was incorrect. The minimum period of follow-up in the study was 24 months.

Frozen (10 μm) sections were mounted onto microscope slides and stained with toluidine blue before microdissection to remove as much normal tissue as possible and ensure a maximum percentage of tumour in each specimen. All tumour specimens used in this study contained more than 50% tumour tissue. Once the dissection was complete samples were digested in 100 μl of lysis buffer (Wright and Manos, 1990). Genomic DNA was extracted from venous blood by lysis with Triton-X100. To examine LOH at THRB, D3S686, D3S32 and D3S30 polymerase chain reaction-restriction fragment length polymorphism (PCR-RFLP) analysis of normal and tumour samples was performed using one or two rounds of PCR analysis (Sundaresan *et al.*, 1992). Amplification was performed in a volume of 50 μl containing 5 μl of DNA solution or 50 ng of genomic DNA. An aliquot of 15 μl of the product was digested with 10 units of the appropriate restriction enzyme. The digests were fractionated on 4% agarose gels, stained with ethidium bromide and photographed.

PCR primers for 13 polymorphic microsatellite markers (see Table I) were obtained from Research Genetics, Huntsville, USA, or synthesised locally. One of the primers was end-labelled with [$\gamma$-$^{32}$P]ATP and PCR products generated from standard reactions. Products were separated by gel electrophoresis in denaturing 8% polyacrylamide-8 M urea and autoradiographed overnight. Labelled M13mp8 was included as a sequencing ladder to facilitate sizing of the alleles. The likely map positions of the markers (see Table I) are given as indicated by the Sigma mapping program using data from the Genome Data Base, The Johns Hopkins University (Naylor *et al.*, 1994).

Matched tumour and normal samples were evaluated subjectively and by computer-assisted quantitative densitometry using Gel Base Pro image software. Allele loss was

**Table I** Polymorphic markers used and LOH at each locus

| *Locus/symbo* | *Map position* | *Allelic loss/ informative cases (%)* |
|---|---|---|
| D3S1307 | 3p26.5 | 2/36 (5) |
| D3S1038 | 3p26.2–25.3 | 5/37 (13) |
| D3S192 | 3p26.1–24.2 | 8/34 (23) |
| D3S1007 | 3p26.1–25.1 | 6/33 (18) |
| D3S1293 | 3p24.3 | 5/33 (15) |
| THRB | 3p24.2 | 10/44 (23) |
| D3S647 | 3p24.1–22.1 | 4/30 (13) |
| D3S32 | 3p22.1–21.2 | 10/28 (36) |
| D3S686 | 3p21.3 | 11/38 (29) |
| D3S966 | 3p21.32–21.31 | 9/38 (24) |
| D3S1076 | 3p22.1–14.2 | 2/29 (7) |
| D3S1228 | 3p14.2–14.1 | 4/31 (13) |
| D3S1079 | 3p13 | 4/25 (16) |
| D3S659 | 3p13 | 6/36 (17) |
| D3S30 | 3p12.3–12.1 | 12/32 (37) |
| D3S196 | 3p27–8 | 3/31 (10) |
| D3S1764 | 3p22–24 | 1/24 (4) |

scored if the signal of one of the alleles was reduced by approximately 50% when DNA from the tumour was compared with normal. Constitutional homozygosity was taken to be non-informative. PCR-based techniques may not distinguish between allele loss and gain, thus alteration in the intensity of alleles is often designated allele imbalance rather than LOH. However, when analysing this series of matched samples we only rarely detected overamplification of one allele with loss or reduced intensity of the matching allele when comparing normal and tumour samples. Furthermore as the alleles showing reduced intensity were within regions considered to harbour tumour-suppressor genes, these results suggest that the allele imbalance detected is likely to be due to LOH. The Mann–Whitney, chi-square and Fisher's exact tests and Spearman correlation coefficients were used for statistical analysis of the results. Survival curves were obtained by the Kaplan–Meier survival method and analysed by the log-rank test.

### Results

A total of 48 primary oral SCCs were screened for LOH at 3p. A summary of the clinical and histological features is given (Table II). The percentage of informative cases with loss at each locus varied between 0% and 37%, (Table I). When all loci were considered the overall LOH for 3p was 34/48 (71%). In this study the frequency of LOH for loci at 3q was 4–10%. This is lower than the incidence of 10–15% random loss reported for other studies of head and neck SCC (Ah-See *et al.*, 1994; Narwoz *et al.*, 1994; Naylor *et al.*, 1994) and may reflect the high percentage of early stage 1 and 2 tumours examined. The frequency of LOH at D3S1307, D3S647 and D3S1076 was below the threshold for random loss at 3q and these loci were considered to lie outside of the regions which may contain suppressor genes.

Examination of the pattern of LOH reveals interstitial losses at three regions at this chromosome arm. Allelic deletion at a single region occurred in 19/48 (40%) cases, 15/48 (31%) showed deletion of two or three regions and 14 cases retained heterozygosity at all informative loci. The likely map positions of the markers (Figure 1a) and the extent of single and multiple deletions is summarised diagrammatically (Figure 1b). The map position of some of the markers used has not been precisely defined and in this schematic representation the order of the markers is shown after taking into account the likely map positions together with the pattern of allelic deletions in the tumours examined. When alleles were lost in the tumour, loss was not always complete owing to contamination of the sample with normal cells. A series of representative cases is shown (Figure 2).

The highest frequency of LOH was seen at 3p24–pter with 18/48 (37%) of combined cases informative at loci between D3S1038 and THRB showing LOH. Cases 5 and 32 show a single interstitial deletion involving THRB suggesting that this locus may be close to a tumour-suppressor gene. The overall pattern of deletions within this region suggests that LOH at 3p24 and 3p25–pter may occur independently. However, as only five cases show allelic deletion at D3S1038 while retaining heterozygosity at D3S1293 or THRB, no firm conclusions can be made until further cases have been analysed and the exact map order of the markers is established.

The second region of deletion was at 3p21.3–22.1 with 17/48 (35%) of combined cases showing allele loss at loci between D3S32 and D3S966. Two cases (22 and 27) showed an interstitial deletion at D3S32. However many of the tumours examined showed deletion at two or more of loci at D3S32, D3S686 and D3S966. Based on the likely map positions of these markers (Figure 1a) this suggests that a tumour-

**Table II** Clinical and histological features of oral SCC

| *Feature* | *Category* | *LOH positive* | *LOH negative* |
|---|---|---|---|
| Age | Average and range | 59.3 (36–84) | 63.9 (34–88) |
| Site | Floor of mouth | 11 | 3 |
| | Alveolus | 10 | 1 |
| | Tongue | 6 | 5 |
| | Retromolar fossa | 2 | 1 |
| | Bucca mucosa | 5 | 3 |
| | Palate | 0 | 1 |
| Sex | Male | 22 | 7 |
| | Female | 12 | 7 |
| Tumour stage | 1 | 3 | 5 |
| | 2 | 11 | 6 |
| | 3 | 9 | 2 |
| | 4 | 11 | 1 |
| Smoking | Non smokers | 16 | 4 |
| | Moderate (<20 $day^{-1}$) | 11 | 6 |
| | Heavy (>20 $day^{-1}$) | 6 | 3 |
| Alcohol | Never | 13 | 8 |
| | Incidental | 6 | 4 |
| | Moderate (1–4 units $day^{-1}$) | 6 | 1 |
| | Heavy (>4 units $day^{-1}$) | 9 | 1 |
| Lymph node status at histology | Positive | 15 | 2 |
| | Negative | 18 | 12 |
| Degree of tumour differentiation | Well | 23 | 10 |
| | Moderate | 9 | 1 |
| | Poor | 2 | 3 |

**Figure 1** (**a**) Map positions of the markers used at 3p as indicated by the Sigma mapping program using data from the Genome Data Base. (**b**) Deletion map of chromosomal regions with partial loss at 3p. The markers used are shown on the left. The order of the markers is given after taking into account the likely map positions together with mapping information provided by the pattern of allelic deletions in the tumours examined. The case numbers are at the top. Cases 1–20 show LOH at one of the commonly deleted regions identified at 3p, cases 21–34 show deletions at two or three of these regions, cases 35–48 retain heterozygosity at all loci tested.

suppressor gene is located close to 3p21.33. Fifteen of 47 (31%) informative combined cases showed interstitial deletions at loci between D3S30 and D3S1228 (3p12.1–14.2) adding a third target for a tumour-suppressor gene close to D3S30.

We compared our results with clinicopathological features for each tumour. Single deletions at 3p12.1–14.2 and 3p21.3–22.1 were more frequent in early stage 1 and 2 tumours whereas multiple losses at 3p12.1–14.2 and 3p21.3–22.1 and 3p24–pter were seen in advanced tumours.

When analysing the results we considered the number of regions showing LOH at 3p rather than the number of deleted loci (as several tumours show loss of adjacent loci within a commonly deleted region). Study of potentially malignant lesions also reveals that deletion at several adjacent loci occur in premalignant lesions (Emilion *et al.*, 1995). There was a significant correlation between the number of deleted regions at 3p and presence of positive lymph nodes at histological examination ($U=137$, $P=0.004$) and tumour stage ($r_s=0.586$, $P<0.001$, Figure 3).

We also compared the presence or absence of LOH at any of the loci examined at 3p (as LOH+/LOH−) to analyse the results in relation to other clinicopathological parameters and known risk factors for oral cancer. LOH+/LOH− at 3p was compared among the three histological groups well-, moderately and poorly differentiated but no correlation was found (chi-square 4.10, $P$0.12) and there was no relation between smoking habits and LOH at 3p (Chi-square 3.87, $P$0.14) or alcohol consumption (chi-square 1.2, $P$0.55, moderate and heavy drinkers were combined for statistical analysis).

We calculated the overall survival time from the date of tumour diagnosis. Analysis of this survival data showed that LOH at 3p is associated with a significant reduction in mean survival time and is a marker of poor prognosis (log-rank 5.13, $P$0.023, Figure 4a). Only three advanced stage 3 or 4 tumours show no LOH at 3p. Separation of the cases into early (stage 1 and 2) and advanced stage tumours identifies a subgroup of patients with aggressive early disease associated with reduced overall survival, (log-rank 5.32, $P$0.021, Figure 4b). LOH at 3p also reduces the disease-free survival time

**Figure 2** Examples of LOH at D3S192 (case 2), D3S32 (case 8), THRB and D3S30 (case 26). All cases retain heterozygosity at the other loci shown. Markers were amplified from DNA derived from blood (N) and tumour (T). The numbering of the patients is the same as used in Table I. ←, alleles detected by microsatellite assay. A1 and A2 represent the polymorphic alleles detected by RFLP analysis. The signal from the smaller second allele is not readily detected in some cases.

(log-rank 4.08, *P* 0.043, Figure 4c). Although these results demonstrate that LOH at 3p correlates with reduced survival, the number of cases examined is insufficient to permit multivariate analysis to see whether this association would remain significant when other known prognostic markers are taken into account.

### Discussion

Our previous studies of LOH at 3p for primary oral SCCs showed allele loss in 81% of informative cases (Partridge *et al.*, 1994). A subsequent study has shown loss in 52% of

**Figure 3** Relationship between tumour stage and the number of commonly deleted regions at 3p. Commonly deleted regions: ■, 3 ▨, 2; ▧, 1; □, 0.

**Figure 4** Influence of LOH at 3p on (**a**) overall survival time for all cases, (**b**) overall survival time for stage 1 and 2 tumours. (**c**) disease-free survival time for all cases.

these tumours and suggests three regions of loss (Wu *et al.*, 1994). In this study the frequency of LOH at 3p was 71% and the data confirms at least three regions of deletion at 3p for oral cancer. Similar results have been suggested for head and neck SCC and other tumour types (Yokota *et al.*, 1989; Jones and Nakamura, 1992; Hibi *et al.*, 1993; Kohono *et al.*, 1993; Maestro *et al.*, 1993; Chen *et al.*, 1994; Wu *et al.*, 1994) and together support the existence of several tumour suppressor genes at chromosome 3p.

Allele loss at 3p12.1–14.2 and 3p21.3–22.1 was seen in early stage 1 and 2 disease suggesting that LOH at these chromosomal regions is an early event in tumour development. The recent finding of LOH at 3p in 53% of potentially malignant oral lesions supports this view (Emilion *et al.*, 1995). Allele loss towards the telomere at 3p24–pter was more common in advanced stage 3 and 4 oral SCC and may be associated with tumour progression. This view is supported by the finding of 56% LOH at 3p26 for a series of head and neck cancers (Li *et al.*, 1994), 94% of the cases in this series were advanced stage 3 or 4 tumours.

Previous deletion mapping studies on 3p have highlighted deletions at 3p24–pter, 3p21.1–23 and 3p12–14 (Yokota *et al.*, 1989 Hibi *et al.*, 1993; Kohono *et al.*, 1993; Maestro *et al.*, 1993; Chen *et al.*, 1994; Partridge *et al.*, 1994). The most commonly deleted region in this study was between 3p24–pter, a finding recently reported for head and neck cancer (Li *et al.*, 1994). The second most frequently deleted region was at 3p21.3–22.1. Earlier studies have also indicated that an area at 3p21–23 is frequently deleted in head and neck and oral cancer (Maestro *et al.*, 1993; Wu *et al.*, 1994). The majority of cases analysed in this series show loss of two or more loci at D3S32, D3S686 and D3S966. The likely chromosomal locations of these markers and the pattern of deletions suggests that a tumour-suppressor gene lies close to D3S21.3. The third commonly deleted region was towards the centromere at 3p12.1–14 with the pattern of deletions suggesting that a suppressor gene also lies close to D3S30.

Several tumour-suppressor genes located at 3p have already been identified and may play a role in the pathogenesis of lung, renal, breast, female genital tract, head and neck and oral cancer. The gene responsible for von Hippel–Lindau syndrome with inherited susceptibility to various neoplasms, is at 3p25 (Shuin *et al.*, 1994) and the retinoic acid receptor $\beta$ gene, which is deleted in several cancer types is at 3p23–4 (Mattei *et al.*, 1988). *PTP-*$\gamma$ (La Forgia *et al.*, 1991) is a candidate suppressor gene recently reassigned to 3p14 and the renal cancer gene *NRC1* also lies within 3p14–12 (Sanchez *et al.*, 1994). Whether the same genes are implicated in all of these tumours or specific suppressor genes exist for some tumour types has yet to be determined. The two cases with interstitial deletions at THRB (case 5 and 32) and the single deletion at D3S32 (case 27) will facilitate mapping of the suppressor genes at the regions identified.

Since the findings are broadly similar to those reported for lung cancer (Hibi *et al.*, 1993) it has been suggested that the common regions of deletion at 3p may be a reflection of the contribution of tobacco to the pathogenesis of these lesions. However this study failed to demonstrate an association with tobacco consumption, (or high alcohol intake, another risk factor for oral cancer) and allele loss at 3p.

The significant correlation between the number of deleted regions at 3p and tumour stage is consistent with the concept that the progressive accumulation of genetic events is associated with advanced disease (Fearon and Vogelstein, 1990). This study has also demonstrated that LOH at 3p is an important marker of reduced survival for early stage oral SCC and identifies a subgroup of patients with aggressive disease who should receive more aggressive treatment. Based on our present knowledge and experience we would advocate wider resection margins and prophylactic treatment of the neck by surgery or radiotherapy to give these patients the best possible chance of long-term survival. Frequent, meticulous follow-up of these cases is also indicated. At present there is no proven adjuvant therapy that can reduce the risk of distant metastasis in patients treated for oral cancer. Prospective studies using the new, better-tolerated retinoid derivatives should be initiated for this subgroup of patients.

**Acknowledgements**

We thank B Carritt, P Rabbitts and V Sundaresan for helpful advice and the provision of oligonucleotides and D Archer, A Brown, G Forman, P Johnson, P Rhys-Evans and G Sockett for access to patients included in this study. This work was supported by a Grant from the Joint Research Committee, King's College Hospital, London.

### References

AH-SEE KW, COKE TG, PICKFORD IR, SOUTAR D AND BALMAIN A. (1994). An allelotype of squamous carcinoma of the head and neck using microsatellite markers. *Cancer Res.*, **54**, 1617–1621.

CARR RJ AND LANGDON JD. (1989). Multiple primaries in mouth cancer. *Br. J. Oral Maxillofacial Surg.*, **27**, 394–399.

CHEN L-C, MATSUMURA K, DENG G, KURISU W, LJUNG B-M, LERMAN MI., WALDMAN FM AND SMITH HS. (1994). Deletion of two separate regions on chromosome 3p in breast cancer. *Cancer Res.*, **54**, 3021–3024.

EMILION G, LANGDON JD, SPEIGHT P AND PARTRIDGE M. (1996). Frequent gene deletions in potentially malignant oral lesions. *Br. J. Cancer.*, (in Press).

FEARON ER AND VOGELSTEIN B. (1990). A genetic model for colorectal tumorigenesis. *Cell*, **61**, 759–767.

HENK JM AND LANGDON JD. (1994). Carcinoma of the oral cavity–management of the primary tumour. In *Malignant Tumours of the Mouth, Jaws and Salivary Glands.* Langdon JD, Henk JM (eds) pp. 154–156. Edward Arnold: London.

HIBI K, TAKAHASHI K, YAMAKAWA K, UEDA R, SEKIDO Y, ARIYOSHI Y, SUYAMA M, TAKAGI H, NAKAMURA Y AND TAKAHASHI T. (1993). Three distinct regions involved in 3p deletion in lung cancer. *Oncogene*, **7**, 445–449.

HIRATA RM, JAQUES DA, CHAMBERS RG, TUTTLE JR AND MAHONEY WE. (1975). Carcinoma of the oral cavity. An analysis of 478 cases. *Ann. Surg.*, **182**, 98–106.

JONES MH AND NAKAMURA Y. (1992). Deletion mapping of chromosome 3p in female genital tract malignancies using microsatellite polymorphisms. *Oncogene*, **7**, 1631–1634.

KOHNO T, TAKAYAMA H, HAMAGUCHI M, TAKANO H, YAMAGUCHI N, TSUDA H, HIROHASHI S, VISSING H, SHIMIZU M, OSHIMURA M AND YOKOTA J. (1993). Deletion mapping of chromosome 3p in human uterine cervical cancer. *Oncogene*, **8**, 1825–1832.

LAFORGIA S, MORSE B, LEVY J, BARNEA G, CANNIZZARO LA, LI F, NOWELL PC, BOGHOSIAN-SELL L, GLICK J., WESTON A, HARRIS CC, DRABKIN H, PATTERSON D, CROCE CM, SCHLESSINGER J AND HUEBNER K. (1991). Receptor protein-tyrosine phosphatase $\gamma$ is a candidate tumour suppressor gene at human chromosome region 3p21. *Proc. Natl Acad. Sci. USA*, **88**, 5036–5040.

LI X, LEE NK, YE Y-W, WABER PG, SCHWEITZER C, CHENG Q-C AND NISEN PD. (1994). Allelic loss at chromosomes 3p, 8p, 13q and 17p associated with poor prognosis in head and neck cancer. *J. Natl Cancer Inst.*, **86**, 1524–1530.

LUNA MA. (1983). Oral communication, quoted by Goopfert H. In, Are we making progress? *Arch. Otolaryngol.*, **110**, 563–564.

MAESTRO R, GASPAROTTO D, VUKOSAVLJEVIC T, BARZAN L, SULFARO S AND BOIOCCHI M. (1993). Three discrete regions of deletion at 3p in head and neck cancers. *Cancer Res.*, **53**, 1–5.

MATTEI M-G, DE THE H, MATTEI JF, MARCHIO A, TIOLLAIS P AND DEJEAN A. (1988). Assignment of the human hap retinoic acid receptor RAR$\beta$ gene to the band p24 of chromosome 3. *Hum. Genet.*, **80**, 188–189.

NARWOZ H, VAN DER REIT P, HRUBAN RH, KOCH W, RUPPERT JM AND SIDRANSKY D. (1994). Allelotype of head and neck squamous cell carcinoma. *Cancer Res.*, **54**, 1152–1155.

NAYLOR SL, BUYS CHCM AND CARRITT B. (1994). Report of the Fourth Single Chromosome Workshop. *Cytogenet. Cell Genet.*, **65**, 1–38.

PARTRIDGE M, KIGUWA S AND LANGDON JD. (1994). Frequent deletion of chromosome 3p in oral squamous cell carcinoma. *Oral Oncol. Eur. J. Cancer*, **30B**, 248–252.

SANCHEZ Y, EL-NAGGAR A, PATHAK S AND MCNEILL KILLARY A. (1994). A tumour suppressor locus within 3p14-p12 mediates rapid cell death of renal cell carcinoma in vivo. *Proc. Natl Acad. Sci. USA*, **91**, 3383–3387.

SHUIN T, KONDO K, TORIGOE S, KISHIDA T, KUBOTA Y, HOSAKA M, NAGASHIMA Y, KITAMURA H, LATIF F, ZBAR B, LERMAN MI AND YAO M. (1994). Frequent somatic mutations and loss of heterozygosity of the von Hippel-Lindau tumour suppressor gene in primary human renal cell carcinomas. *Cancer Res.*, **54**, 2852–2856.

SUNDARESAN V, GANLY P, HASLETON P, RUDD R, SINHA G, BLEEHAN NM AND RABBITTS P. (1992). p53 and chromosome 3 abnormalities, characteristic of malignant lung tumours, are detectable in pre-invasive lesions of the bronchus. *Oncogene*, **7**, 1989–1997.

WRIGHT DK AND MANOS MM. (1990). In *PCR Protocols: a Guide to Methods and Applications*, Wright DK and Manos MM (eds) pp. 152–158, Academic Press: San Diego.

WU CL, SLOAN P, READ AP, HARRIS R AND THAKKER N. (1994). Deletion mapping on the short arm of chromosome 3 in squamous cell carcinoma of the oral cavity. *Cancer Res.*, **54**, 6484–6488.

YOKOTA J, TSUKADA Y, NAKAJIMA T, GOTOH M, SHIMOSATO Y, MORI N, TSUNOKAWA Y, SUGIMURA T AND TERADA M. (1989). Loss of heterozygosity on the short arm of chromosome 3 in carcinoma of the uterine cervix. *Cancer Res.*, **49**, 3598–3601.